# Rheumatology in East Asia

**DOI:** 10.1186/s13075-018-1542-y

**Published:** 2018-03-23

**Authors:** Kazuhiko Yamamoto, Yeong-Wook Song, Zhan-guo Li

**Affiliations:** 1 RIKEN Center for Integrative Medical Sciences, 1-7-22 Suehiro-cho, Tsurumi-ku, Yokohama, Kanagawa 230-0045 Japan; 2 Seoul National University Medical Research Center, Seoul, South Korea; 3 Peking University People’s Hospital, Peking, China

**Keywords:** Japan, Korea, China, rheumatology, JCR, KCR, CRA, APLAR, EAGOR

## Abstract

Europe and North America have been leaders in rheumatology for many years. However, for more than a decade now the East Asian region has been catching up dramatically. Some aspects of rheumatology in East Asia are now almost comparable to those in the European League Against Rheumatism (EULAR) and the American College of Rheumatology (ACR). In this article, we describe recent progress in rheumatology in East Asia, focusing specifically on Japan, Korea, and China.

## Background

This manuscript aims to contribute to the series entitled ‘Rheumatology around the world’.

## General situation of rheumatology in East Asia

Japan is one of the four countries that established the South East Asia and Pacific Area League Against Rheumatism in 1963, which has now been renamed the Asia Pacific League of Association for Rheumatology (APLAR). The Japan College of Rheumatology (JCR) has nearly 10,000 members and about 4500 registered rheumatology specialists. More than 6000 attend each annual meeting of the JCR.

In South Korea, the first rheumatology symposium was held in 1981. Thereafter, in 1992, a new subspecialty board system was established. The rheumatologists certified by the Korean College of Rheumatology (KCR) has increased to more than 300. Similarly, Chinese rheumatology has developed rapidly over the past 20 years. In 2008, about 2600 rheumatologists were reported by the Chinese Rheumatology Association (CRA) and, in 2014, this figure had risen to over 5000 rheumatologists.

Apart from their domestic rheumatology meetings, APLAR meetings have recently been held annually and have become important opportunities to sustain communication and discussion. Furthermore, in 1997 the Korea-Japan Combined Meeting of Rheumatology (KJCMR) was started which was then expanded to include China as the East Asia Group of Rheumatology (EAGOR) in 2005. In this way, rheumatologists in East Asia have been getting together to communicate and improve themselves through friendly rivalry.

## Clinical aspects of rheumatology

Pharmaceutical administration and regulations in Japan were rather conservative. For example, methotrexate (MTX) was approved for rheumatoid arthritis (RA) in 1999, which was more than 10 years later than in Western countries. However, the system in Japan has improved, and approval intervals have been shortened over the past several years. One of the most characteristic administrative policies on biological therapies in Japan is compulsory investigation of all cases treated with a newly approved biological drug. According to this regulation, all RA patients, several thousand to nearly 10,000, who received an original biological drug were registered and safety data for the initial 6 months were reported. These procedures have strengthened clinical rheumatology in Japan. Several important clinical findings were reported from studies during and after these clinical investigations [1].

Anti-tumor necrosis factor (TNF) biologic treatment for RA was started in Korea in 2000. In 2011, the KCR Biologic Registry (KOBIO) was launched to monitor clinical effects, as well as adverse events, in patients treated with biologic agents.

On the other hand, the CRA published management guidelines for 23 rheumatic diseases to direct practice in China. However, there still exists heterogeneity in clinical practice.

## Basic research in rheumatology

Japan has been one of the leaders in basic immunology, with discoveries of several cytokines such as invterleukin (IL)-6 [2], regulatory T cells, and signals in innate immunity. On the other hand, clinical immunology including rheumatology research has not been as strong as basic immunology, although interesting findings have been regularly published from Japan.

Since 2000, Korean rheumatologists have joined innovative government research projects. Several important findings have been published, including antigen-specific T cells in RA and synovial cells [3]. The osmoprotective transcription factor nuclear factor of activated T cells (NFAT)5, which regulates macrophage survival by inducing CCL2 secretion, was found to enhance chronic arthritis by conferring apoptotic resistance to activated macrophages [4]. Copy number of leukocyte-specific protein (LSP)1 was significantly lower in RA patients suggesting its role in the pathogenesis of RA by promoting migration of T cells into the target tissues [5].

Chinese rheumatologists have been also contributing greatly. For example, TNF-induced FOXP3 dephosphorylation was reported in regulatory T cells in RA [6]. He et al. reported a novel circulating CCR7^lo^PD-1^hi^ follicular helper T cell (Tfh), which was correlated with clinical indices and autoantibody production in RA and systemic lupus erythematosus (SLE) [7]. Zhu et al. showed that IL-17 contributes to autoimmune pathogenesis by suppressing miR-23b expression in resident cells and promoting proinflammatory cytokine expression in patients with SLE and RA [8]. A study showed that the oral and gut microbiomes were perturbed in RA and were partly normalized after treatment [9]. Furthermore, treatment with low-dose recombinant human IL-2 was reported to selectively modulate the numbers of regulatory T cells, Tfhs, and IL-17-producing helper T cells with marked reductions of disease activity of SLE [10].

Genetic studies in rheumatology in East Asia are rather advanced. Before the current genome-wide association studies (GWAS), several leading GWASs were published from Japan [11, 12]. Furthermore, researchers in East Asia joined the project of trans-ethnic meta-analysis of RA GWASs [13], and important papers were subsequently published from Japan, Korea, and China. Studies of SLE and Behçet’s disease have also been extensively performed. One GWAS in the Han population identified two new susceptibility loci for ankylosing spondylitis [14]. In addition, a GWAS in Han Chinese identified a susceptibility locus for primary Sjögren’s syndrome at 7q11.23 [15]. Thus, strong genetic studies are now conducted in this region. Moreover, functional genomic and epigenetic studies have also been conducted by several researchers in this region [16, 17].

In conclusion, rheumatology in East Asia has been dramatically developing in several fields in both clinical and basic aspects.

## Abbreviations

ACR: American College of Rheumatology
APLAR: Asia Pacific League of Association for Rheumatology
CCL: C-C motif chemokine ligand
CCR: C-C motif chemokine receptor
CRA: Chinese Rheumatology Association
EAGOR: East Asia Group of Rheumatology
EULAR: European League Against Rheumatism
FOXP3: Forkhead box P3
GWAS: Genome-wide association studies
IL: Interleukin
JCR: Japan College of Rheumatology
KCR: Korean College of Rheumatology
KJCMR: Korea-Japan Combined Meeting of Rheumatology
KOBIO: Korean College of Rheumatology Biologic Registry
LSP: Leukocyte-specific protein
miR: MicroRNA
MTX: Methotrexate
NFAT: Nuclear factor of activated T cells
PD: Programmed cell death
RA: Rheumatoid arthritis
SLE: Systemic lupus erythematosus
Tfh: Follicular helper T cells
TNF: Tumor necrosis factor
